# Physical activity, functional capacity, and mental health during the first postoperative month in patients undergoing ambulatory pulmonary wedge resection: a prospective observational study

**DOI:** 10.1186/s12890-026-04481-y

**Published:** 2026-07-08

**Authors:** Qiaoling Wang, Zhongxing Bing, Shuai Wei, Xiaojie Wang, Bo Yu, Riwang Wang, Lixia Chen, Lu Zhang

**Affiliations:** 1 Department of Rehabilitation Medicine, Peking Union Medical College Hospital, Chinese Academy of Medical Sciences and Peking Union Medical College, Beijing, 100730 China; 2 Department of Thoracic Surgery, Peking Union Medical College Hospital, Chinese Academy of Medical Sciences and Peking Union Medical College, Beijing, 100730 China

**Keywords:** Day-case VATS wedge surgery, Actigraph accelerometers, Step counts, Physical activity, Mental health

## Abstract

**Background:**

As a well-established clinical model, ambulatory surgery has been routinely performed in thoracic surgery. Accordingly, this study aimed to investigate home-based physical activity, functional capacity, mental health in patients undergoing ambulatory VATS for lung cancer.

**Method:**

This study prospectively observed the changes in physical activity, functional capacity, mental health and quality of life on 1 month after surgery, with a comparison between the postoperative and preoperative to identify the indicators associated with early postoperative mobility, and further screened out the potential preoperative predictors for postoperative mobility. Among 150 patients assessed for eligibility, 97 were enrolled, 82 underwent day-case VATS wedge resection, and 72 had complete 4-week follow-up data and constituted the complete-case analytic cohort.

**Result:**

A total of 72 patients underwent day-case VATS, were discharged within 23 h postoperatively, and completed the 4-week follow-up. The cohort had a mean age of 50.4 ± 12.0 years, with 88.9% stageⅠA1 and 11.1% stageⅠA2 lung cancer. The compliance of wearing Actigraph accelerometers was satisfactory within 4 weeks after surgery. During the 4-week follow-up, patients showed a significant upward trend in daily step count, mean daily METs, and moderate-intensity physical activity time (all *P* < 0.05), while light-intensity activity decreased notably from week 1 to week 2 and remained stable thereafter. No significant changes were observed in sedentary time across the 4 weeks. Compared with the preoperative baseline, significant decreases were observed in 6MWD(*P* = 0.043), MEP(*P* = 0.001), mMRC grade(*P* = 0.044) and EQ-VAS score(*P* = 0.002), whereas SF-12 score(*P* = 0.003) increased significantly. Correlation analysis showed week 1 step count correlated significantly with preservation of exercise capacity (Δ6MWT) (*r* = 0.369, *P* = 0.035). Univariate linear regression identified higher GAD-7 scores were strongly associated with lower activity levels (*P* = 0.02). Safety outcomes were analyzed in the all-operated cohort (*n* = 82, primary safety population) and the pathology-confirmed malignant cohort (*n* = 78, secondary safety population). Two patients (2.44%, 95% CI 0.30–8.53%) experienced postoperative complications requiring 30-day readmission- for pulmonary infection. No 30-day reoperation or mortality was observed (0/82, 95% CI 0–4.40%).

**Conclusion:**

Patients undergoing day-case VATS wedge resection showed a weekly increase in home-based activity, reaching near physical activity levels of normal healthy individuals by week 4 without full recovery of preoperative physical capacity. The mean daily step count at week 1 was correlated with functional performance at week 4. Preoperative anxiety level was inversely associated with physical activity within the first postoperative week, indicating that higher preoperative anxiety corresponded to lower postoperative physical activity level.

**Supplementary Information:**

The online version contains supplementary material available at https://doi.org/10.1186/s12890-026-04481-y.

## Introduction

Lung cancer is among the most prevalent cancers and a leading cause of cancer mortality worldwide, China included [1]. Concurrently, the affected population is getting younger, and many previously healthy individuals are found to have suspected lung cancer in routine screenings [2]. The field of thoracic surgery has undergone remarkable transformation. For selected lung resection procedures, besides conventional inpatient elective lung resections, day surgery protocols for lung resection have been gradually implemented [3, 4]. Day surgery for lung resection is defined as patient discharge within 23 h postoperatively, and the transition to this model has been facilitated by advances in minimally invasive techniques—most notably the evolution of video-assisted thoracoscopic surgery (VATS) [5, 6]. Enhanced Recovery After Surgery (ERAS) protocols have further accelerated this process, with up to 37% of patients now being safely discharged within 18 h following minimally invasive anatomic lung resection [7, 8]. This transition toward day surgery has completely reshaped the perioperative care model, and as rehabilitation practitioners, we also aim to explore the functional recovery status of this surgical population after they return home from the hospital.

Previous studies on day surgery for lung resection have indicated that patients may experience early postoperative functional decline, such as in pulmonary function, respiratory muscle strength, and exercise tolerance [9, 10]. These symptoms usually gradually improve within 3–6 months after surgery, but full recovery of pulmonary function may take 6–12 months [11, 12]. The present study is designed to observe the physical acitivity, functional capacity of patients who underwent day-case lung wedge resection at 1 month postoperatively. Walking is a core component of physical activity and daily step count is widely recognized as an effective indicator for quantifying physical status [13]. Sufficient evidence has confirmed an association between postoperative step count and postoperative functional outcomes [14]. Given that step count data are usually collected via patient self-report—a method prone to recall bias—this study uses wearable devices to objectively track patients’ daily step counts within 1 month after surgery.

In the diagnosis and treatment of lung cancer, routine assessment of health-related quality of life (HRQoL) has been incorporated into clinical guideline recommendations [15]. At present, the HRQoL outcomes of patients who underwent day surgery for ambulatory wedge resection at 1 month postoperatively remain unclear, which is also a key focus of observation in this study.

Therefore, the primary objectives of this study are as follows: to investigate the recovery of physical activity, functional capacity, and mental health in patients who underwent day surgery for ambulatory wedge resection after 1 month, as well as the changes compared with their preoperative status; and to identify relevant preoperative indicators that can predict patients’ functional status at 1 month postoperatively.

## Patients and methods

### Study design

This study is an analysis of a prospective observational trial. The study protocol has been reviewed and approved by the Ethics Committee of Peking Union Medical College Hospital (approval number: I-24PJ1720).

### Study population

**Fig. 1:**
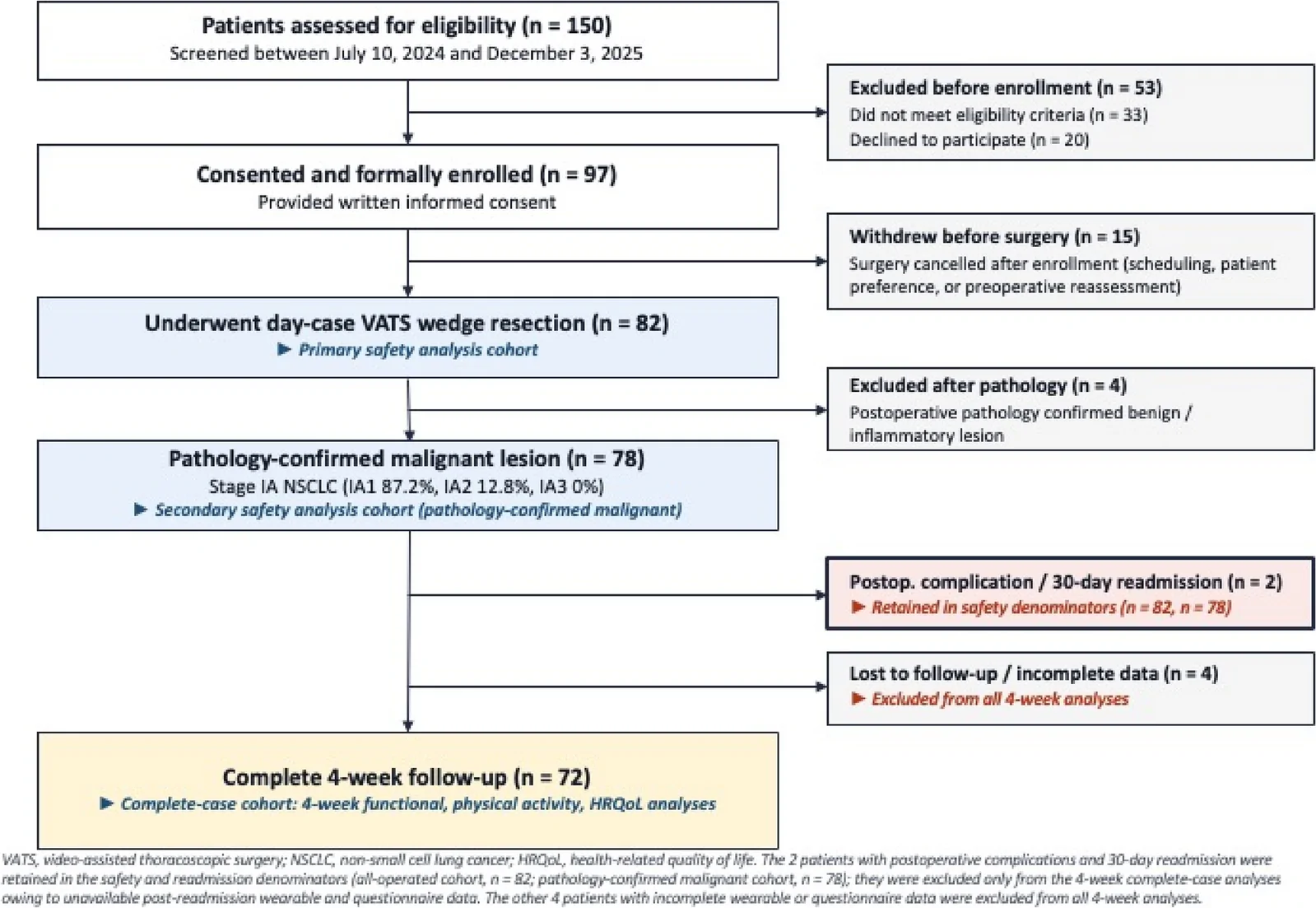
Patient flow diagram

Eligibility was assessed in 150 consecutive patients scheduled for elective day-case VATS wedge resection between July 2024 and December 2025. Inclusion criteria were aged 18–75 years, normal cognitive function with ability to understand and follow study instructions, and ability to read and comprehend the local language. Prespecified pre-enrollment exclusion criteria were severe cognitive impairment or psychiatric disorders, severe visual or auditory impairments affecting device usage, and inability to use or learn electronic device operation. Of the 150 patients screened, 53 were excluded before enrollment (33 not meeting inclusion criteria; 20 declined participation), and 97 patients provided written informed consent and were formally enrolled. Subsequent post-enrollment events (cancelled surgery, *n* = 15; benign or inflammatory pathology, *n* = 4) were transparently reported in Fig. 1 and were not part of the prespecified eligibility criteria but rather post-enrollment losses. 82 patients underwent the scheduled day-case VATS wedge resection. 78 patients had pathologically confirmed malignant lesions, and 72 had complete 4-week wearable and questionnaire follow-up data.

Three analysis cohorts were defined. The complete-case cohort (*n* = 72), comprising patients with valid 4-week follow-up data, was used for analyses of physical activity, functional capacity, mental health, and HRQoL. The all-operated cohort (*n* = 82, primary) served as the denominator for primary safety analyses, with all operated patients retained regardless of follow-up completeness or complication status and the pathology-confirmed malignant cohort (*n* = 78, secondary), with the 2 patients who experienced postoperative complications and 30-day readmission retained in both safety denominators. The pathology-confirmed malignant cohort (*n* = 78) was used for sensitivity analyses to validate the robustness of primary findings.

### Wearable-based physical activity tracking

Upon providing informed consent, participants were required to wear an ActiGraph wrist-worn accelerometer (ActiGraph wGT3X-BT, USA) to track their physical activity for 1 month after surgery. The device has a battery life of over 1 month, eliminating the need for participants to recharge it. Daily wear time was recorded for each participant, with a valid monitoring day defined as no less than 10 h of daily wear time to ensure data reliability, consistent with established accelerometer protocols [16, 17].

### Data collection and outcome measures

All assessments were conducted by trained research personnel using standardized protocols at baseline (pre-surgery) and one month post-operatively.

The clinical data of each patient were collected one week before surgery after anesthesia evaluation and confirmation of the surgical date. Supplementary data were extracted from medical records, including age, gender, weight, and height. The study also recorded health-related variables such as smoking history, drinking history, and comorbidities (e.g., hypertension, diabetes mellitus, cardiovascular diseases, chronic obstructive pulmonary disease [COPD]).

Physical activity levels were objectively measured using Actigraph wrist-worn accelerometers for 1 month postoperatively recording metabolic equivalents (METs), daily step counts, and duration of low-intensity, moderate-intensity, and moderate-to-high intensity activities.

Physical fitness parameters included grip strength assessed using a Jamar hydraulic hand dynamometer (Model 5030J1, Jamar Technologies, Hatfield, PA, USA). Grip strength testing was performed with participants seated, shoulder adducted, elbow flexed at 90°, and forearm in neutral position. Three maximal contractions were recorded for each hand with 30-second rest intervals, and the highest value was used for analysis. Maximal inspiratory pressure (MIP) and maximal expiratory pressure (MEP) were measured via portable pulmonary function testing (FGC-A+, Ankang Medical Equipment, China). Respiratory muscle strength testing was conducted with participants seated upright, using a flanged mouthpiece with nose clips. Participants performed maximal inspiratory efforts from residual volume for MIP and maximal expiratory efforts from total lung capacity for MEP, with three trials performed and the highest sustainable pressure for one second recorded. Six-minute walk test (6MWT) distance was assessed using standardized protocols along a 30-meter indoor corridor. Quadriceps strength was evaluated through isometric muscle testing using a BIODEX isokinetic dynamometer (BIODEX Medical Systems, Shirley, NY, USA). Participants were positioned with hip flexed at 90° and knee flexed at 60°, with the dynamometer axis aligned with the lateral femoral condyle. Three maximal voluntary isometric contractions were performed with 60-second rest periods, and peak torque values were normalized to body weight.

Psychological and sleep assessments employed validated questionnaires including the Generalized Anxiety Disorder 7-item scale (GAD-7) [18], Patient Health Questionnaire-9 (PHQ-9) [19], and Pittsburgh Sleep Quality Index (PSQI) [20]. Health-related quality of life (HRQoL) was evaluated using the 12-item Short Form Health Survey (SF-12) [21] and self-reported health status via the EuroQol Five-Dimension Five-Level scale(EQ-5D5L) [22] and the EuroQol Visual Analog Scale (EQ-VAS) [23].

### Ethics statement

This study was conducted in accordance with the Declaration of Helsinki and International Conference on Harmonisation Good Clinical Practice guidelines. All participants provided written informed consent after receiving comprehensive information regarding study objectives, procedures, potential risks, and benefits. Participants retained the right to withdraw from the study at any time without affecting their standard medical care. All patient data were maintained with strict confidentiality and used exclusively for research purposes, with participant privacy protected through anonymization procedures during data processing.

### Statistical analysis

**Fig. 2:**
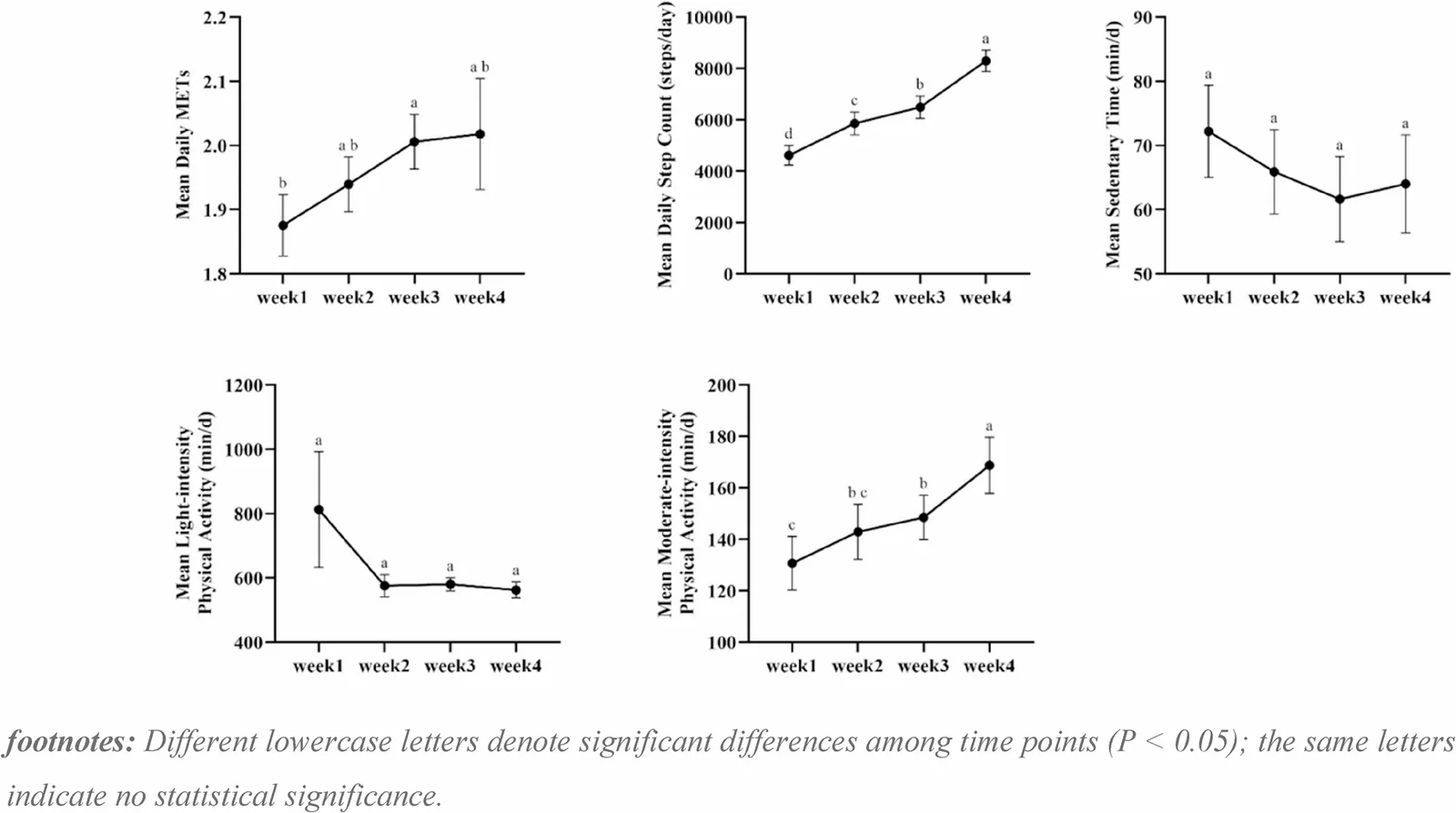
Temporal trends in physical activity parameters during the 4-week postoperative period

All statistical analyses were performed using SPSS 26.0 software, and a two-sided P value < 0.05 was considered statistically significant. The normality of continuous variables was assessed using the Kolmogorov–Smirnov test. Continuous variables were presented as mean ± standard deviation if normally distributed or as median (interquartile range(IQR)) if non-normally distributed. Categorical variables were summarized as frequencies and percentages. For between-group comparisons, normally distributed continuous variables were analyzed using the independent-samples t test, whereas non-normally distributed variables were analyzed using the Mann–Whitney U test. Categorical variables were compared using the chi-square test or Fisher’s exact test when expected cell counts were less than 5. Temporal changes in postoperative physical activity indicators over the 4-week follow-up period were analyzed using one-way repeated-measures ANOVA when the assumptions for parametric testing were met. Spearman’s rank correlation analysis was used to assess associations between Week 1 daily step count and changes in functional outcomes. Univariate linear regression analysis was performed to explore baseline predictors of early postoperative physical activity.To assess robustness against potential attrition and selection bias, four prespecified sensitivity analyses were performed: (i) baseline comparison between the 72 complete-case and 6 non-complete-case patients; (ii) worst-case and best-case single-value imputation for the primary 4-week endpoints; (iii) multiple imputation (m = 20) under a missing-at-random assumption with baseline covariates as predictors; and (iv) descriptive re-analysis of perioperative variables in the all-operated cohort. Results of the sensitivity analyses are reported in Supplementary Tables S2–S4. Fig. 2 was generated using GraphPad Prism 9.0.

## Results

### Study flow

Between July 10, 2024 and December 3, 2025, 150 patients were assessed for eligibility (Fig. 1). Fifty-three were excluded before enrollment (33 not meeting inclusion criteria; 20 declined). Ninety-seven patients were enrolled. Fifteen had surgery cancelled before the operative date, leaving 82 who underwent day-case VATS wedge resection (all-operated cohort). Of these, 4 had benign or inflammatory pathology, leaving 78 with pathology-confirmed malignant disease. Six of the 78 patients did not contribute complete 4-week follow-up data: 2 due to postoperative complications and 30-day readmission (retained in safety/readmission denominators) and 4 due to incomplete wearable or questionnaire data. The final complete-case cohort for 4-week physical activity, functional, mental health, and HRQoL analyses comprised 72 patients.

### General participant characteristics

A total of 78 patients undergoing VATS wedge resection were enrolled in the present study, including 72 patients with complete 4 week follow-up, 2 patients developed postoperative complications or 30-day readmission and 4 patients were lost to follow-up or had incomplete data (Table 1).

**Table 1:** Baseline characteristics of patients

Baseline characteristics	Pathology-confirmed malignant cohort (*n* = 78)
Male, n(%)	16 (20.5%)
Age, years	50.4 ± 12.0
BMI, kg/m^2^	24.1 ± 3.5
Current smoker, n(%)	8 (10.3%)
ASA physical status, n(%)	
I	32 (41.0%)
II	46 (59.0%)
Educational level, n(%)	
High school or college diploma	26 (33.3%)
Bachelor’s degree	30 (38.5%)
Graduate degree	22 (28.2%)
Employment status, n(%)	
Employed	48 (61.5%)
Retired	28 (35.9%)
Student	2 (2.6%)
Comorbidity, n(%)	
Depression	5 (6.4%)
Hypertension	10 (12.8%)
Asthma	2 (2.6%)
History of thyroid cancer	4 (5.1%)
History of endometrial cancer	2 (2.6%)
Coronary heart disease	2 (2.6%)
Preoperative	
FEV1%pre	103.1 ± 12.8
FVC %pre	109.6 ± 14.4
Location of tumor, *n* (%)	
Left	30 (38.5%)
Right	48 (61.5%)
VATS wedge resection, *n* (%)	78 (100%)
Stage I A(T1N0M0), *n* (%)	
I A1	68 (87.2%)
I A2	10 (12.8%)
I A3	0 (0.0%)

Values are presented as Mean±SD for normally distributed variables and Median (IOR) for non-normally distributed variables. Baseline characteristics of patients with complete 4 week follow-up(n=72) was shown in the Table S1

*BMI* body mass index, *ASA physical status* American Society of Anesthesiologists physical status, *FEV1* forced expiratory volume in 1 second, *FVC* forced vital capacity, *VATS* video-assisted thoracoscopic surgery

The mean age of the overall cohort was 50.4 ± 12.0 years, and the mean BMI was 24.1 ± 3.5 kg/m^2^. There were 16 male patients (20.5%) and 8 current smokers (10.3%). Most participants were classified as ASA Ⅱ physical status (59.0%). Hypertension was the most common comorbidity (12.8%), followed by depression (6.4%), while other comorbidities were rare.

Preoperative pulmonary function showed that the predicted FEV1% was 103.1 ± 12.8% and predicted FVC% was 109.6 ± 14.4%. Tumors were located in the right lung in 61.5% of patients and in the left lung in 38.5% of patients. All patients received VATS wedge resection. All cases were stage ⅠA (T1N0M0), including 68 cases of ⅠA1 (87.2%) and 10 cases of ⅠA2 (12.8%), with no ⅠA3 patients.

Baseline demographic, clinical, pulmonary function and pathological characteristics were comparable between patients with complete follow-up (*n* = 72) and those with incomplete follow-up (*n* = 6). No significant differences were found in all baseline variables between the two groups (all *P* > 0.05) (Table S1), indicating balanced baseline distribution and low risk of selection bias related to missing follow-up data.

### Safety analysis

Safety outcomes were analyzed in the all-operated cohort (*n* = 82, primary safety population) and the pathology-confirmed malignant cohort (*n* = 78, secondary safety population). Two patients (2.44%, 95% CI 0.30–8.53%) experienced postoperative complications requiring 30-day readmission- for pulmonary infection. No 30-day reoperation or mortality was observed (0/82, 95% CI 0–4.40%). These two patients were retained in the safety and readmission denominators in accordance with intention-to-treat-like principles, and were excluded only from the 4-week complete-case analyses (*n* = 72) because of unavailable post-readmission wearable and questionnaire data, as prespecified in the Statistical Analysis section (Table 2).

**Table 2:** Safety outcomes in the prespecified primary (all-operated, *n* = 82) and secondary (pathology-confirmed malignant, *n* = 78) safety analysis cohorts

Safety outcome	All-operated cohort (*n* = 82)		Pathology-confirmed malignant cohort (*n* = 78)	
	*n* (%)	95% CI	*n* (%)	95% CI
Successful same-day discharge (≤ 23 h)	82 (100.00%)	95.60–100.00	78 (100.00%)	95.38–100.00
Postoperative complications	2 (2.44%)	0.30–8.53	2 (2.56%)	0.31–8.96
30-day all-cause readmission	2 (2.44%)	0.30–8.53	2 (2.56%)	0.31–8.96
30-day reoperation	0 (0.00%)	0.00–4.40	0 (0.00%)	0.00–4.62
30-day all-cause mortality	0 (0.00%)	0.00–4.40	0 (0.00%)	0.00–4.62

Data are presented as n (%) with two-sided exact 95% confidence intervals (Clopper–Pearson method). The all-operated cohort (*n* = 82) was the prespecified primary safety analysis population; the pathology-confirmed malignant cohort (*n* = 78) was the prespecified secondary safety analysis population. In accordance with intention-to-treat–like principles, the 2 patients who experienced postoperative complications and 30-day readmission were retained in both safety denominators and were excluded only from the 4-week complete-case functional, physical activity, and health-related quality of life analyses (*n* = 72) owing to unavailable post-readmission wearable and questionnaire data. No formal between-cohort hypothesis testing is reported because the two cohorts are nested (the *n* = 78 cohort is a subset of the *n* = 82 cohort)

*VATS* video-assisted thoracoscopic surgery, *CI* confidence interval

### Actigraph watch wearing compliance and physical activity during postoperative week1 recovery

#### Analyses below are based on the complete-case cohort (*n* = 72)

Actigraph accelerometer wearing compliance over the 4-week postoperative period is presented in Table 3. The total expected wearing days per week were 504 (72 patients × 7 days/week).

**Table 3:** Actigraph accelerometer wearing compliance during the 4-week postoperative period

	Week1	Week2	Week3	Week4
Expected wearing days^a^	504	504	504	504
Actual wearing days	456 (90.5%)	456 (90.5%)	420 (83.3%)	396 (78.6%)
Valid wearing days^b^	440 (87.3%)	436 (86.5%)	400 (79.4%)	372 (73.8%)
Days included in analysis^c^	432 (85.7%)	428 (84.9%)	386 (76.6%)	354 (70.2%)
Patient with adequate compliance^d^	64 (88.9%)	64 (88.9%)	60 (83.3%)	56 (77.8%)

^a^Expected wearing days = 72 patients × 7 days per week

^b^Valid wearing days were defined as days with ≥10 hours of wear time [16, 17]

^c^Days included in analysis represents the total number of valid wearing days from patients who met the weekly compliance criterion (≥4 valid days per week)

^d^Patients with adequate compliance were defined as those with ≥4 valid wearing days per week

In postoperative Week 1, the actual wearing days were 456 (90.5%), valid wearing days (≥ 10 h/day) were 440 (87.3%), days included in analysis were 432 (85.7%), and 64 patients (88.9%) met adequate compliance (≥ 4 valid days/week). Week 2 showed consistent compliance: actual and valid wearing rates remained 90.5% and 86.5%, respectively, with 88.9% of patients achieving adequate compliance.

Compliance gradually declined over time. In Week 3, actual wearing compliance decreased to 83.3%, valid compliance to 79.4%, days included in analysis to 76.6%, and 60 patients (83.3%) had adequate compliance. By Week 4, further reductions were observed: actual wearing 78.6%, valid wearing 73.8%, analysis days 70.2%, and 56 patients (77.8%) with adequate compliance.

Overall, accelerometer wearing compliance was satisfactory during the 4-week postoperative period, with a mild time-dependent decline. Over 70% of valid data were retained in Week 4, indicating good feasibility and patient acceptability of the monitoring protocol in the postoperative rehabilitation phase.

Table 4 presents the longitudinal changes in objectively assessed physical activity levels across the first four weeks following day-case lung resection.Overall, MET levels showed a gradual upward trend from week 1 to week 3 and remained stable at week 4 (1.88 ± 0.28 to 2.01 ± 0.45). Daily step count increased progressively throughout the four postoperative weeks, with continuous rises in mean, median, minimum and maximum step values. Daily sedentary time fluctuated slightly but showed a mild declining trend in mean values over time. Light-intensity physical activity was highest in week 1 and decreased markedly thereafter, staying relatively stable from week 2 to week 4. Moderate-intensity activity and total MVPA (moderate-to-vigorous physical activity) increased steadily across the four weeks, while vigorous-intensity activity remained zero at all postoperative time points.

**Table 4:** Longitudinal changes in objectively measured physical activity during the first 4 weeks after day-case lung resection

Physical Activity Variable	Week1	Week2	Week3	Week4
METs	1.88 ± 0.28	1.92 ± 0.25	2.01 ± 0.24	2.01 ± 0.45
Daily Step Count				
Mean ± SD	4616 ± 2200	5858 ± 2556	6518 ± 2426	8299 ± 2184
Median(IQR)	4486(2946,5940)	5415(3828, 7766)	6397(4925,8085)	8824(6516,9801)
Minimum	1359	1493	2491	4453
Maxmum	10,845	13,028	15,273	13,726
Sedentary Time, min/d				
Mean ± SD	75.7 ± 38.6	69.6 ± 29.9	71.8 ± 30.0	69.7 ± 30.8
Median(IQR)	69.3(40.4, 96.0)	66.0(40.1,91.0)	63.6(38.1,80.0)	60.0(35.6, 76.8)
Minimum	32.3	33.0	31.0	30.1
Maxmum	166.2	167.5	154.9	132.0
Light-intensity activity, min/d	812 ± 132	575 ± 199	580 ± 115	562 ± 130
Moderate-intensity activity, min/d	131 ± 60	143 ± 62	149 ± 48	169 ± 57
Vigorous-intensity activity, min/d	0	0	0	0
Total MVPA, min/d	131 ± 60	143 ± 62	149 ± 48	169 ± 57

Values are presented as Mean±SD for normally distributed variables and Median (IOR) for non-normally distributed variables

*MVPA* Moderate-to-Vigorous Physical Activity

Dynamic changes in physical activity parameters over the 4-week postoperative period are presented in Fig. 2. Paired comparisons of measurements at weeks 1, 2, 3, and 4 revealed that: Mean daily METs, daily step count, and moderate-intensity physical activity time increased significantly over time (*p* < 0.05), with different superscript letters in the figure indicating statistically significant differences between weeks; Light-intensity physical activity time decreased sharply from week 1 to week 2 (*p* < 0.05) and remained stable thereafter; Sedentary time showed a gradual downward trend, but no statistically significant differences were found between any two weeks (*p* > 0.05).

### Functional outcomes and health-related quality of life

#### Analyses below are based on the complete-case cohort (*n* = 72)

From baseline to 4 weeks postoperatively (Table 5), exercise capacity was found to be significantly reduced in the 6-minute walk test (6MWT: 572.89 ± 70.76 to 548.05 ± 72.95 m, − 4.3%, *P* = 0.043). Maximal expiratory pressure declined significantly (107.56 ± 22.56 to 97.89 ± 22.79cmH₂O, − 9.0%, *P* = 0.001), while maximal inspiratory pressure showed a nonsignificant trend (− 4.2%, *P* = 0.139). Peripheral muscle strength was preserved (handgrip: −1.4%/-2.9%, *P* = 0.346/0.050; quadriceps: −2.5%/-0.6%, *P* = 0.704/0.944). Dyspnea increased significantly (mMRC grade1ཞ2 from 5.56% to 30.56%, *P* = 0.044). Anxiety scores showed a non-significant decreasing trend (GAD-7: 3.0(2.0,5.0) to 2.0(0.0,5.0), *P* = 0.070), and depression scores showed no significant change (PHQ-9: 3.0(1.0,4.0) to 2.0(1.0,4.0), *P* = 0.722).

**Table 5:** Changes in functional outcomes and health-related quality of life from baseline to 4 weeks after day-case lung resection

Outcome Measure	Baseline	Week 4	∆	*P*
Functional capacity				
6MWT, m	572.89 ± 70.76	548.05 ± 72.95	-24.84 ± 71.06	0.043^*^
Respiratory muscle strength				
MIP, cmH₂O	81.33 ± 24.33	77.96 ± 24.25	-3.38 ± 10.83	0.139
MEP, cmH₂O	107.56 ± 22.56	97.89 ± 22.79	-9.67 ± 11.74	0.001^*^
Peripheral muscle strength				
Grip, kg				
left	25.15 (23.00, 28.53)	23.85 (21.65, 29.80)	-0.35	0.346
Right	27.30 (24.00, 32.05)	25.85 (23.75, 31.13)	-0.78	0.050
Quadriceps strength, kg				
left	33.95 (29.08, 43.68)	32.75 (27.95, 39.25)	-0.85	0.704
Right	36.10 (28.63, 42.40)	36.00 (29.93, 44.45)	-0.20	0.944
Patient-reported outcomes				
mMRC dyspnea scale				0.044^*^
0	68 (94.44.%)	50 (69.44%)		
1	4 (5.56%)	20 (27.78%)		
2	0 (0.00%)	2 (2.78%)		
GAD-7 anxiety score	3.00 (2.00, 5.00)	2.00 (0.00, 5.00)	-1.00	0.070
PHQ-9 depression score	3.00 (1.00, 4.00)	2.00 (1.00, 4.00)	0.00	0.722
PSQI sleep quality score	6.00 (4.00, 8.25)	7.00 (4.00, 10.00)	1.00	0.087
Health-related quality of life				
EQ-5D5L value set	1.00 (0.95, 1.00)	1.00 (0.89, 1.00)	0.00	0.063
EQ-VAS	90.00 (85.00, 95.00)	85.00 (80.00, 90.00)	-5.00	0.002^*^
SF-12	76.25 (67.50, 84.06)	82.50 (71.41, 89.38)	4.69	0.003^*^

Values are presented as Mean±SD for normally distributed variables and Median (IOR) for non-normally distributed variables

* indicates statistically significant difference (*p*< 0.05)

*6MWT* 6-minute walk test, *MIP* maximal inspiratory pressure, *MEP* maximal expiratory pressure, *mMRC* modified Medical Research Council, *GAD-7* Generalized Anxiety Disorder-7, *PHQ-9* Patient Health Questionnaire-9, *PSQI* Pittsburgh Sleep Quality Index, *EQ-5D5L* EuroQol 5 Dimensions 5 Levels, *EQ-VAS* EuroQol Visual Analogue Scale, *SF-12* 12-Item Short Form Health Surve*y*

Quality of life measures showed divergent patterns: EQ-VAS decreased (90(85,95) to 85(80,90), − 5.6%, *P* = 0.002), while SF-12 improved substantially (76.25(67.50,84.06) to 82.50(71.41,89.38), + 6.2%, *P* = 0.003), exceeding the minimal clinically important difference. EQ-5D5L value set showed a nonsignificant trend (*P* = 0.063).

**Fig. 3:**
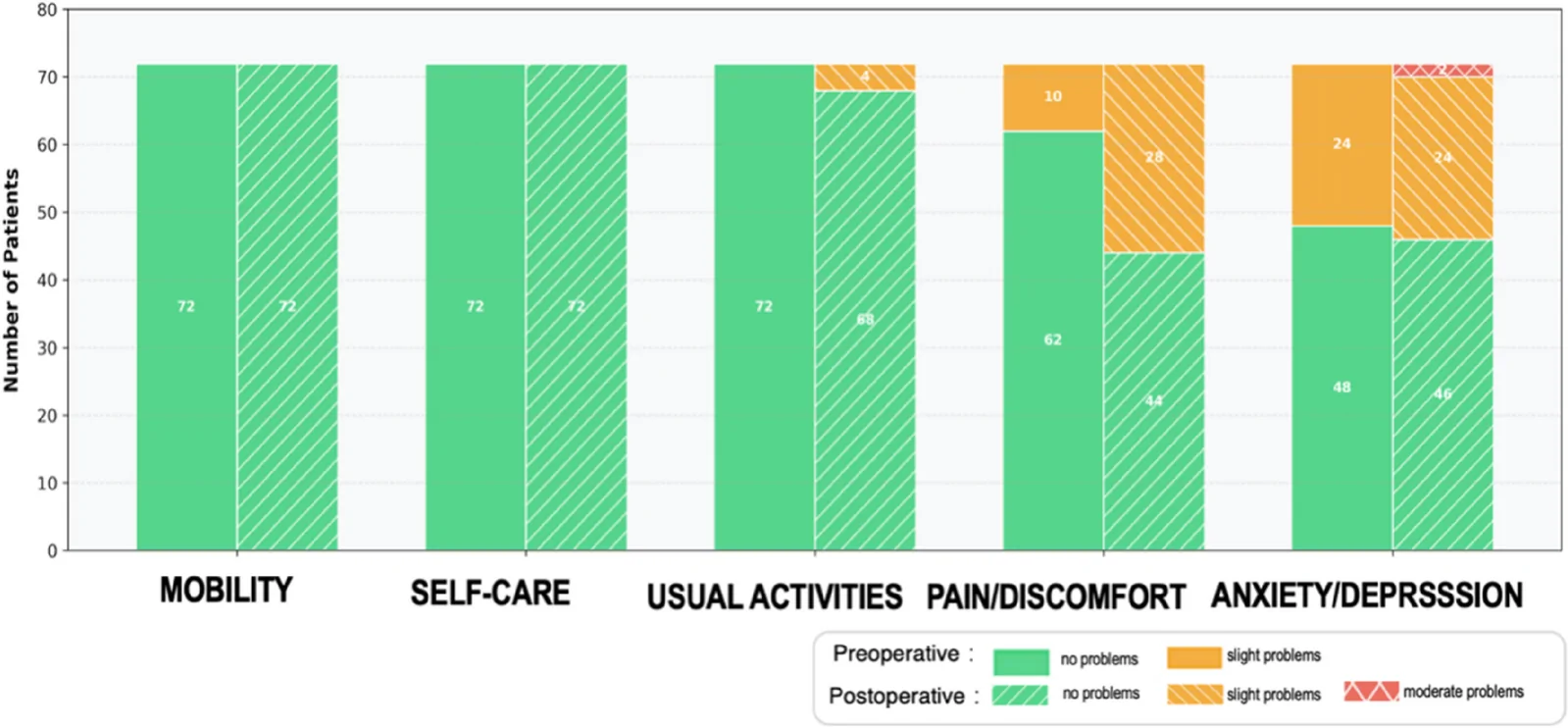
Preoperative vs. postoperative EQ-5D-5 L score distribution in 72 patients

The changes in EQ-5D-5 L dimensions of mobility, self-care, and usual activities remained stable, whereas those in the dimension of pain/discomfort and anxiety/depression showed marked variability (Fig. 3). Overall, functional capacity and respiratory-specific measures declined modestly, peripheral strength were maintained, and psychological outcomes and health-related quality of life improved substantially.

### Correlation between early postoperative daily step count and changes in functional outcomes

#### Analyses below are based on the complete-case cohort (*n* = 72)

**Fig. 4:**
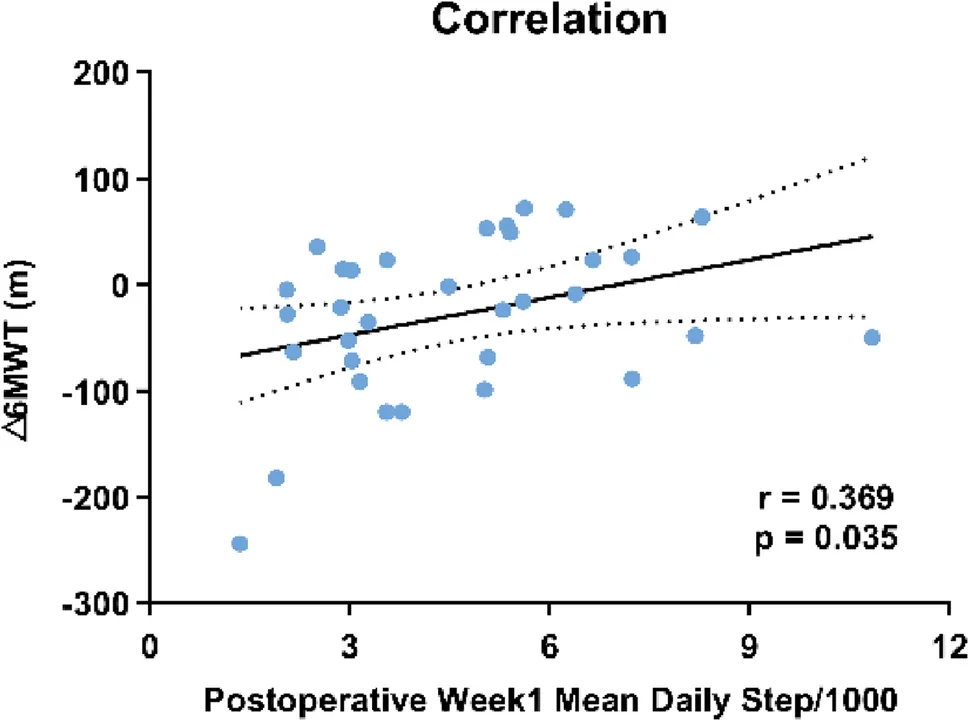
Correlation between mean daily step count during postoperative week 1 and Δ6MWT at 4 weeks after day-case lung resection

We examined correlations between mean daily step count during postoperative week 1 and changes in functional outcomes from baseline to week 4 (Table 6, Fig. 4). Week 1 step count correlated significantly with preservation of exercise capacity (6-minute walk test: Spearman’s rho = 0.369, *P* = 0.035). No significant correlations were observed with respiratory muscle strength (MIP: rho = − 0.157, *P* = 0.383; MEP: rho = − 0.02, *P* = 0.913), psychological outcomes (GAD-7: rho = 0.077, *P* = 0.669; PHQ-9: rho = 0.140, *P* = 0.437), or quality of life measures (EQ-VAS: rho = 0.047, *P* = 0.796; SF-12: rho = 0.024, *P* = 0.896). These findings suggest early activity levels predict physical functional preservation but not psychological recovery, supporting the use of week 1 step count as an early indicator of functional trajectory and potential tool for identifying patients requiring intensified rehabilitation.

**Table 6:** Correlation between mean daily step count at postoperative week 1 and changes in functional outcomes (multiple imputation, *n* = 20 datasets)

Variable	Patients with complete follow-up (*n* = 72)			Multiple imputation pooled (*n* = 20 datasets))		
	Spearman’s rho	95% CI	*P*-value	Spearman’s rho	95% CI	*P*-value
Δ6MWT, m	0.369	0.019 to 0.639	0.035*	0.261	0.235 to 0.369	< 0.001^*^
ΔMIP, cm H₂O	-0.157	-0.483 to 0.207	0.383	-0.124	-0.196 to 0.051	0.070
ΔMEP, cm H₂O	-0.020	-0.370 to 0.913	0.913	-0.015	-0.088 to 0.059	0.688
ΔGrip, kg						
ΔLeft	0.057	-0.302 to 0.402	0.753	0.044	-0.30 to 0.117	0.232
ΔRight	0.146	-0.218 to 0.474	0.419	0.133	-0.060 to 0.014	0.057
ΔQuadriceps strength, kg						
ΔLeft	0.086	-0.275 to 0.426	0.632	0.068	-0.006 to 0.141	0.063
ΔRight	-0.164	-0.488 to 0.200	0.362	-0.135	-0.207 to 0.062	0.349
ΔGAD-7	0.077	-0.283 to 0.419	0.669	0.060	-0.014 to 0.133	0.102
ΔPHQ-9	0.140	-0.224 to 0.469	0.437	0.149	-0.076 to0.220	0.050
ΔPSQI	-0.211	-0.525 to 0.153	0.238	-0.198	-0.268 to 0.026	0.098
ΔEQ5D5L	0.198	-0.166 to 0.515	0.268	0.163	-0.091 to 0.234	0.056
ΔEQ-VAS	0.047	-0.311 to 0.393	0.796	0.065	-0.009 to 0.138	0.075
ΔSF-12	0.024	-0.332 to 0.373	0.896	0.003	-0.071 to 0.077	0.934

Multiple imputation was performed using SPSS 26.0 to handle missing 4-week follow-up data, generating 20 imputed datasets. All variables in the table, as well as baseline indicators correlated with postoperative week 1 mean daily step count and 4-week outcomes, were included as predictors. Correlation analyses were conducted separately in each imputed dataset, and pooled results were synthesized using Rubin’s rules

* indicates statistically significant difference (*p*< 0.05)

*6MWT* 6-minute walk test, *MIP* maximal inspiratory pressure, *MEP* maximal expiratory pressure, *mMRC* modified Medical Research Council, *GAD-7* Generalized Anxiety Disorder-7, *PHQ-9* Patient Health Questionnaire-9, *PSQI* Pittsburgh Sleep Quality Index, *EQ-5D5L* EuroQol 5 Dimensions 5 Levels, *EQ-VAS* EuroQol Visual Analogue Scale, *SF-12* 12-Item Short Form Health Survey, *CI* confidence interval

Meanwhile, sensitivity analysis was performed using multiple imputation. The effect directions and statistical significance were consistent with those of the complete-case analysis, confirming the robustness of the primary findings.

### Baseline predictors of early postoperative activity

#### Analyses below are based on the complete-case cohort (*n* = 72)

To explore the correlation between preoperative baseline levels and the mean daily step count during the first postoperative week, this study adopted univariate linear regression analysis to analyze potential preoperative baseline influencing factors, and the results are shown in the Table 7. Univariate regression analysis indicated that among all the included factors, only the preoperative GAD-7 score was statistically significantly associated with the mean daily step count during the first postoperative week (β=-0.45, 95%CI: -0.94~-0.09, *P* = 0.02), suggesting that the higher the preoperative GAD-7 score, the fewer the mean daily steps of patients during the first postoperative week, presenting a negative correlation. The P-values of the other factors were all > 0.05.

**Table 7:** Univariate linear regression analysis of baseline factors associated with mean daily step count during postoperative week 1

**Variable **	**β-value**	**95% CI**	***P-*** **value**
Gender	-0.12	-3.16 to 1.73	0.55
Age	0.06	-0.08 to 0.11	0.77
BMI	-0.083	-0.35 to 0.23	0.69
Current smoker	-0.17	-4.20 to 1.79	0.41
Educational level	-0.08	-1.53 to 1.07	0.71
Employment status	0.05	-1.78 to 2.29	0.79
Comorbidity Present	0.20	-1.03 to 2.97	0.33
6MWT before surgery	0.25	-0.01 to 0.03	0.22
Grip before surgery	0.04	-0.11 to 0.13	0.86
GAD-7 before surgery	-0.45	-0.94 to -0.09	0.02^*^
PHQ-9 before surgery	-0.36	-0.91 to 0.05	0.07
PSQI before surgery	-0.31	-0.61 to 0.08	0.12
EQ5D5L before surgery	0.099	-22.426 to 33.305	0.69
EQ-VAS before surgery	0.144	-0.091 to 0.168	0.14
SF-12 before surgery	0.022	-0.075 to 0.083	0.914

*BMI* body mass index, *6MWT* 6-minute walk test, *GAD-7* Generalized Anxiety Disorder-7, *PHQ-9* Patient Health Questionnaire-9, *PSQI* Pittsburgh Sleep Quality Index, *CI* confidence interval, *EQ-5D5L* EuroQol 5 Dimensions 5 Levels, *EQ-VAS* EuroQol Visual Analogue Scale

* indicates statistically significant difference (*p*< 0.05)

## Discussion

A total of 72 patients with complete 4-week follow-up data were included in the final analysis. The study cohort was characterized by a mean age of 50.6 ± 12.0 years, a relatively high proportion of female patients, a predominance of ASA Ⅱ physical status (58.3%), and a high proportion of stage ⅠA1 lung cancer (88.9%), which is consistent with the demographic and pathological characteristics of patients undergoing day-case lung resection in clinical practice [24, 25]. These participants exhibited generally good baseline health status, a favorable level of pulmonary function, and optimal physical fitness. None of the patients developed pulmonary complications such as pneumonia within one month after surgery, and all patients maintained ambulatory activity during the one-month home period. The balanced baseline characteristics between the complete follow-up group and the incomplete follow-up group (all *P* > 0.05) effectively reduced the selection bias caused by missing follow-up data, ensuring the reliability of the study results.

Additionally, the study cohort demonstrated excellent postoperative compliance with wrist-worn device wear, which exceeded our expectations. Correspondingly, the weekly postoperative step count of the participants was also higher than anticipated. Data collected from wearable devices showed that within 4 weeks after surgery, the patients’ weekly average MET value, average daily step count, and average daily duration of moderate-to-vigorous physical activity increased progressively week by week. By the 4th week, the patients’ weekly mean MET value reached 2.01 ± 0.45, equivalent to the physical activity intensity of household chores; their mean daily step count reached 8299 steps, which was close to the range for healthy adults (7000–10000 steps/day). Previous studies have also demonstrated that earlier hospital discharge followed by a home transition period after surgery is associated with a better home transition experience [26]. We postulate that the absence of a thoracic drainage tube likely mitigated intercostal nerve stimulation, thereby enhancing patients’ willingness to engage in ambulation during home-based recovery [27, 28]. This may partially account for the higher-than-expected weekly step count observed after surgery.

Although the daily physical activity level of patients could recover to that of healthy adults at 1 month after surgery, their mean 6MWD and MEP were still significantly lower than the preoperative values. This indicates that the cardiopulmonary endurance and other components of physical fitness had not yet fully returned to the preoperative level, which is consistent with the findings of previous studies [9, 11, 12]. In practice, during postoperative 6MWT and respiratory muscle strength assessments, some patients failed to fully cooperate and exert their maximum effort to complete the tests, due to concerns that brisk walking and forceful inspiration and expiration might pull at their surgical incisions. Consequently, the test results obtained on this occasion may have underestimated patients’ actual functional capacity, as they were confounded by the influence of patients’ subjective effort.

In the self-reported scales: mMRC grade at 1 month after surgery was higher than that before surgery, demonstrating that patients were more prone to dyspnea during physical activity at this time point; EQ-VAS score was also lower than the preoperative baseline, suggesting that patients subjectively perceived a decline in their health status compared with the preoperative state. EQ-5D-5 L results revealed that pain and discomfort at 1 month postoperatively might be the primary cause of patients’ subjective dissatisfaction.

Previous studies in hospitalized lung surgery populations have demonstrated that higher mean daily step count during the first postoperative week is associated with better preservation of exercise capacity [29, 30]. The present study similarly found that mean daily step count during postoperative week 1 was significantly and positively correlated with the change in 6MWT distance from baseline to week 4 (Spearman’s rho = 0.369, *P* = 0.035). Active early mobilization may facilitate the recovery of respiratory muscle function and lower limb strength, as well as the maintenance of cardiopulmonary endurance, thereby contributing to superior functional outcomes. These findings indicate that early physical activity holds important rehabilitative value in patients undergoing day-case minimally invasive lung surgery, and that early mean daily step count may serve as a valuable indicator for predicting subsequent functional recovery trajectory.

Univariate linear regression analysis showed that preoperative anxiety (GAD-7 score) was the only significant predictor of early postoperative daily step count (β=−0.45, *P* = 0.02), and higher anxiety scores were significantly associated with lower early activity levels. Preoperative anxiety is a common emotional problem in perioperative period, with an incidence rate of 25%ཞ80%. Patients with anxiety often have worries about surgical safety, postoperative pain and complications, which leads to reduced activity willingness and decreased activity level after surgery, thus affecting the progress of rehabilitation.

This study has several limitations. First, our 4-week functional and HRQoL analyses were performed as a complete-case analysis on 72 patients with valid follow-up data; 6 patients (2 with postoperative complications/readmission, 4 with incomplete wearable or questionnaire data) were excluded from these specific analyses but retained where appropriate in safety analyses. Baseline characteristics were comparable between the 72 complete-case and 6 non-complete-case patients (Supplementary Table S1-S2), and four prespecified sensitivity analyses (Supplementary Tables S3–S4) confirmed the robustness of our primary findings to plausible assumptions about the missing 4-week data. Second, the relatively small single-center sample (*n* = 72) limits result generalizability; Third, further multicenter large-sample prospective studies are required for validation. the 4-week follow-up is short, and the long-term trajectories of physical activity, functional recovery and HRQoL after day-case VATS wedge resection remain unclear, warranting longer follow-up to observe long-term recovery outcomes.

## Conclusion

Patients undergoing day-case VATS wedge resection for stage ⅠA NSCLC showed a gradual improvement in physical activity levels within 4 weeks after surgery, with satisfactory compliance of objective activity monitoring. Early postoperative daily step count (Week 1) can be used as an early indicator to predict the preservation of exercise capacity, and preoperative anxiety is the key predictor of early postoperative activity. These findings suggest that clinical staff should strengthen the monitoring of early postoperative physical activity, carry out targeted psychological intervention for patients with preoperative anxiety, and formulate individualized rehabilitation plans to promote the comprehensive recovery of patients.

## Supplementary Information

Supplementary Material 1.

## Data Availability

The datasets used and/or analysed during the current study are available from the corresponding author on reasonable request.

## Abbreviations

VATS: Video-assisted Thoracoscopic Surgery
ERAS: Enhanced Recovery After Surgery
HRQoL: Health-related Quality of Life
NSCLC: Non-Small Cell Lung Cancer
COPD: Chronic Obstructive Pulmonary Disease
METs: Metabolic Equivalents
MIP: Maximal Inspiratory Pressure
MEP: Maximal Expiratory Pressure
6MWT: Six-Minute Walk Test
GAD-7: Generalized Anxiety Disorder 7-Item Scale
PHQ-9: Patient Health Questionnaire-9
PSQI: Pittsburgh Sleep Quality Index
SF-12: 12-Item Short Form Health Survey
EQ-5D-5L: EuroQol Five-Dimension Five-Level Scale
EQ-VAS: EuroQol Visual Analog Scale
FEV1: Forced Expiratory Volume in 1 s
FVC: Forced Vital Capacity
BMI: Body Mass Index
mMRC: modified Medical Research Council
MVPA: Moderate-to-Vigorous Physical Activity
