# Supplementary material for: Physical activity, functional capacity, and mental health during the first postoperative month in patients undergoing ambulatory pulmonary wedge resection: a prospective observational study
Source: BMC Pulm Med. 2026 Jul 8;26:443. doi: 10.1186/s12890-026-04481-y (PMC13628754; doi:10.1186/s12890-026-04481-y)
Supplement: Supplementary file 1 [file 12890_2026_4481_MOESM1_ESM.docx]

**Supplementary Materials**

**Table S1 Baseline characteristics of participants with complete follow-up (n=72) and incomplete follow-up data (n=6)**

| ***Baseline characteristics*** | **Patients with complete follow-up** | **Patients with incomplete follow-up data** | ***P-*value** |
| --- | --- | --- | --- |
|  | **(n=72)** | **(n=6)** |  |
| **Male, n**(%) | 14 (19.4%) | 2 (33.3%) | 0.597 |
| **Age, years** | 50.6±12.0 | 47.0±12.6 | 0.476 |
| **BMI, kg/m^2^** | 24.1±3.5 | 24.7±3.6 | 0.707 |
| **Current smoker, n**(%) | 8 (11.1%) | 0 (0.0%) | 1.000 |
| **ASA physical status, n**(%) |  |  | 1.000 |
| **** Expression is faulty **** | 30 (41.7%) | 2 (33.3%) |  |
| **** Expression is faulty **** | 42 (58.3%) | 4 (66.7%) |  |
| **Educational level, n**(%)  High school or college diploma | 24 (33.3%) | 2 (33.3%) | 0.949 |
| Bachelor's degree | 28 (38.9%) | 2 (33.3%) |  |
| Graduate degree | 20 (27.8%) | 2 (33.3%) |  |
| **Employment status, n**(%) |  |  | 0.836 |
| Employed | 44 (61.1%) | 4 (66.7%) |  |
| Retired | 26 (36.1%) | 2 (33.3%) |  |
| Student | 2 (2.8%) | 0 (0.0%) |  |
| **Comorbidity, n**(%) |  |  | 0.705 |
| Depression | 4 (5.6%) | 1 (16.7%) |  |
| Hypertension | 10 (13.9%) | 0 (0.0%) |  |
| Asthma | 2 (2.8%) | 0 (0.0%) |  |
| History of thyroid cancer | 4 (5.6%) | 0 (0.0%) |  |
| History of endometrial cancer | 2 (2.8%) | 0 (0.0%) |  |
| Coronary heart disease | 2 (2.8%) | 0 (0.0%) |  |
| **Preoperative** |  |  |  |
| FEV1 %pre | 102.8±13.3 | 106.1±4.9 | 0.050 |
| FVC %pre | 109.4±15.1 | 111.3±6.0 | 0.057 |
| **Location of tumor, n (%)** |  |  | 1.000 |
| Left | 28 (38.9%) | 2 (33.3%) |  |
| Right | 44 (61.1%) | 4 (66.7%) |  |
| **Stage I A(T1N0M0), n (%)** |  |  | 1.000 |
| **I A1** | 64 (88.9%) | 6 (100%) |  |
| **I A2** | 8 (11.1%) | 0 (0.0%) |  |
| **I A3** | 0 (0.0%) | 0 (0.0%) |  |

*Values are presented as Mean*±*SD for normally distributed variables and Median (IOR) for non-normally distributed variables. BMI, body mass index; ASA physical status, American Society of Anesthesiologists physical status; FEV1, forced expiratory volume in 1 second; FVC, forced vital capacity; VATS: video-assisted thoracoscopic surgery.*

**Table S2. Comparison of preoperative Functional Outcomes and Health-Related Quality of Life between participants with complete follow-up (n=72) and incomplete follow-up (n=6)**

|  | **Patients with complete follow-up** | **Patients with incomplete follow-up data** | ***P-*value** |  |
| --- | --- | --- | --- | --- |
|  | **(n=72)** | **(n=6)** |  |  |
| **Functional capacity** |  |  |  |  |
| 6MWT, m | 572.89±70.76 | 573.67±75.70 | 0.822 |  |
| **Respiratory muscle strength** |  |  |  |  |
| MIP, cmH₂O | 81.33±24.33 | 82.33±26.40 | 0.409 |  |
| MEP, cmH₂O | 107.56±22.56 | 121.67±30.44 | 0.245 |  |
| **Peripheral muscle strength** |  |  |  |  |
| Grip, kg |  |  |  |  |
| left | 25.15 (23.00, 28.53) | 27.1 (25.10, 30.30) | 0.078 |  |
| Right | 27.30 (24.00, 32.05) | 33.00 (26.00, 35.10) | 0.052 |  |
| Quadriceps strength, kg |  |  |  |  |
| left | 33.95 (29.08, 43.68) | 36.60 (31.80, 62.10) | 0.084 |  |
| Right | 36.10 (28.63, 42.40) | 40.60 (30.20, 55.10) | 0.099 |  |
| **Patient-reported outcomes** |  |  |  |  |
| mMRC dyspnea scale |  |  | 1.000 |  |
| 0 | 68 (94.44%) | 6 (100.0%) |  |  |
| 1 | 4 (5.56%) | 0 (0.00%) |  |  |
| 2 | 0 (0.00%) | 0 (0.00%) |  |  |
| GAD-7 anxiety score | 3.00 (2.00, 5.00) | 2.00 (0.00, 5.00) | 0.403 |  |
| PHQ-9 depression score | 3.00 (1.00, 4.00) | 3.00 (0.00, 5.00) | 0.970 |  |
| PSQI sleep quality score | 6.00 (4.00, 8.25) | 6.00 (2.00, 8.00) | 0.596 |  |
| **Health-related quality of life** |  |  |  |  |
| EQ-5D5L value set | 1.00 (0.95, 1.00) | 0.95 (0.89, 1.00) | 0.094 |  |
| EQ-VAS | 90.00 (85.00, 95.00) | 90.00 (85.00, 95.00) | 1.000 |  |
| SF-12 | 76.25 (67.5, 84.06) | 76.25 (56.88, 81.25) | 0.548 |  |

*Values are presented as Mean*±*SD for normally distributed variables and Median (IOR) for non-normally distributed variables. 6MWT, 6-minute walk test; MIP, maximal inspiratory pressure; MEP, maximal expiratory pressure; mMRC, modified Medical Research Council; GAD-7, Generalized Anxiety Disorder-7; PHQ-9, Patient Health Questionnaire-9; PSQI, Pittsburgh Sleep Quality Index; EQ-5D5L, EuroQol 5 Dimensions 5 Levels; EQ-VAS, EuroQol Visual Analogue Scale; SF-12, 12-Item Short Form Health Survey; CI, confidence interval.*

**Table S3. Correlation Between Mean Daily Step Count at Postoperative Week 1 and Changes in Functional Outcomes (Worst-case imputation (n=78, P5) & Best-case imputation (n=78, P95))**

|  | **Patients with complete follow-up(n=72)** | | **Worst-case imputation (n=78, P5)** | | **Best-case imputation (n=78, P95)** | |
| --- | --- | --- | --- | --- | --- | --- |
|  | **Spearman's rho** | ***P-*value** | **Spearman's rho** | ***P-*value** | **Spearman's rho** | ***P-*value** |
| **Δ6MWT, m** | 0.369 | 0.035 | 0.514 | 0.001 | 0.489 | 0.002 |
| **ΔMIP, cm H₂O** | -0.157 | 0.383 | 0.085 | 0.621 | 0.081 | 0.641 |
| **ΔMEP, cm H₂O** | -0.020 | 0.913 | 0.184 | 0.283 | 0.185 | 0.281 |
| **ΔGrip, kg** |  |  |  |  |  |  |
| **ΔLeft** | 0.057 | 0.753 | 0.254 | 0.135 | 0.238 | 0.162 |
| **ΔRight** | 0.146 | 0.419 | 0.328 | 0.051 | 0.315 | 0.061 |
| **ΔQuadriceps strength, kg** |  |  |  |  |  |  |
| **ΔLeft** | 0.086 | 0.632 | 0.282 | 0.095 | 0.264 | 0.120 |
| **ΔRight** | -0.164 | 0.362 | 0.061 | 0.722 | 0.072 | 0.675 |
| **ΔGAD-7** | 0.077 | 0.669 | 0.261 | 0.123 | 0.281 | 0.097 |
| **ΔPHQ-9** | 0.140 | 0.437 | 0.318 | 0.059 | 0.325 | 0.053 |
| **ΔPSQI** | -0.211 | 0.238 | 0.047 | 0.787 | 0.050 | 0.770 |
| **ΔEQ5D5L** | 0.198 | 0.268 | 0.379 | 0.061 | 0.383 | 0.057 |
| **ΔEQ-VAS** | 0.047 | 0.796 | 0.232 | 0.173 | 0.239 | 0.161 |
| **ΔSF-12** | 0.024 | 0.896 | 0.231 | 0.176 | 0.231 | 0.175 |

*6MWT, 6-minute walk test; MIP, maximal inspiratory pressure; MEP, maximal expiratory pressure; mMRC, modified Medical Research Council; GAD-7, Generalized Anxiety Disorder-7; PHQ-9, Patient Health Questionnaire-9; PSQI, Pittsburgh Sleep Quality Index; EQ-5D5L, EuroQol 5 Dimensions 5 Levels; EQ-VAS, EuroQol Visual Analogue Scale; SF-12, 12-Item Short Form Health Survey; CI, confidence interval.*

**Table S4 Baseline characteristics of participants with complete follow-up (n=72) and incomplete follow-up or non-completers (n=10)**

| ***Baseline characteristics*** | **Patients with complete follow-up** | **Patients with incomplete follow-up or non-completers** | ***P-*value** |
| --- | --- | --- | --- |
|  | **(n=72)** | **(n=10)** |  |
| **Male, n**(%) | 14 (19.4%) | 4 (40.0%) | 0.215 |
| **Age, years** | 50.6±12.0 | 43.4±10.6 | 0.054 |
| **BMI, kg/m^2^** | 24.1±3.5 | 24.3±3.5 | 0.820 |
| **Current smoker, n**(%) | 8 (11.1%) | 0 (0.0%) | 0.336 |
| **ASA physical status, n**(%) |  |  | 0.302 |
| **** Expression is faulty **** | 30 (41.7%) | 2 (20.0%) |  |
| **** Expression is faulty **** | 42 (58.3%) | 8 (80.0%) |  |
| **Educational level, n**(%)  High school or college diploma | 24 (33.3%) | 2 (20.0%) | 0.579 |
| Bachelor's degree | 28 (38.9%) | 4 (40.0%) |  |
| Graduate degree | 20 (27.8%) | 4 (40.0%) |  |
| **Employment status, n**(%) |  |  | 0.600 |
| Employed | 44 (61.1%) | 8 (80.0%) |  |
| Retired | 26 (36.1%) | 2 (20.0%) |  |
| Student | 2 (2.8%) | 0 (0.0%) |  |
| **Comorbidity, n**(%) |  |  | 0.474 |
| Depression | 4 (5.6%) | 0 (0.0%) |  |
| Hypertension | 10 (13.9%) | 2 (20.0%) |  |
| Asthma | 2 (2.8%) | 0 (0.0%) |  |
| History of thyroid cancer | 4 (5.6%) | 0 (0.0%) |  |
| History of endometrial cancer | 2 (2.8%) | 0 (0.0%) |  |
| Coronary heart disease | 2 (2.8%) | 0 (0.0%) |  |
| **Preoperative** |  |  |  |
| FEV1 %pre | 102.83±13.31 | 97.5±12.2 | 0.269 |
| FVC %pre | 109.41±15.05 | 103.3±11.6 | 0.269 |
| **Location of tumor, n (%)** |  |  | 0.212 |
| Left | 28 (38.9%) | 2 (20.0%) |  |
| Right | 44 (61.1%) | 8 (80.0%) |  |

*Values are presented as Mean*±*SD for normally distributed variables and Median (IOR) for non-normally distributed variables. BMI, body mass index; ASA physical status, American Society of Anesthesiologists physical status; FEV1, forced expiratory volume in 1 second; FVC, forced vital capacity; VATS: video-assisted thoracoscopic surgery.*
